# Clinical effect of minimally invasive surgery for inguinal cryptorchidism

**DOI:** 10.1186/s12893-020-01010-4

**Published:** 2021-01-06

**Authors:** Yunjin Wang, Liu Chen, Xu Cui, Chaoming Zhou, Qing Zhou, Zhengmian Zhang

**Affiliations:** Department of Pediatric Surgery, Fujian Maternity and Child Health Hospital, Affiliated Hospital of Fujian Medical University, Fuzhou, 350001 People’s Republic of China

**Keywords:** Minimally invasive surgery, Transscrotal incision, Inguinal incision, Laparoscopy, Orchiopexy

## Abstract

**Background:**

The purpose of this study was to investigate the clinical effect of minimally invasive surgery for inguinal cryptorchidism.

**Methods:**

The patients were divided into the minimally invasive surgery group (n = 100) and the traditional surgery group (n = 58). In the minimally invasive surgery group, patients with low inguinal cryptorchidism (n = 54) underwent surgery with a transscrotal incision, and patients with high inguinal cryptorchidism (n = 46) underwent laparoscopic surgery.

**Results:**

There was no difference in the hospital stay duration or cost between the minimally invasive surgery group and the traditional surgery group (P > 0.05). As for the operative time, minimally invasive surgery of low inguinal cryptorchidism was shorter than traditional surgery (P = 0.033), while minimally invasive surgery of high inguinal cryptorchidism was comparable to traditional surgery (P = 0.658). Additionally, there were no cases of testicular atrophy, testicular retraction, inguinal hernia or hydrocele in either group. There was no significant difference in the incidence of poor wound healing between the two groups (*P* > 0.05). Although there was no significant difference in the incidence of scrotal hematoma between the two groups (*P* > 0.05), the incidence in the minimally invasive surgery group was higher than that in the traditional surgery group.

**Conclusions:**

Minimally invasive surgery including a transscrotal incision for low inguinal cryptorchidism and laparoscopic surgery for high inguinal cryptorchidism is as safe and effective as traditional surgery, and could also provide a good cosmetic effect for children.

## Background

Cryptorchidism is one of the most common genital malformations in children. There is no other generally agreed treatment for cryptorchidism than surgery [1, 2]. After Annandale successfully performed orchiopexy in 1879 [3], orchiopexy through an inguinal incision became the classic method for the treatment of inguinal cryptorchidism; however, this operation leaves obvious surgical scars in the groin, which affects a patient’s appearance.

With the medical improvements, minimally invasive surgery has become a developmental trend. Various minimally invasive procedures are performed to treat inguinal cryptorchidism to meet patients’ aesthetic requirements. Laparoscopic orchiopexy has been the gold standard for the treatment of abdominal cryptorchidism and can also be performed for inguinal cryptorchidism to achieve an aesthetic effect [4–10]. Orchiopexy via a single scrotal incision can also be performed for inguinal cryptorchidism to improve the appearance [11–14]. This article retrospectively analysed the clinical data and summarized the experience of minimally invasive surgery for inguinal cryptorchidism.

## Methods

### Patients

We retrospectively analysed the clinical data of 158 patients with inguinal cryptorchidism at our hospital from January 2017 to January 2018. According to the surgical method, the patients were divided into two groups: the minimally invasive surgery group (n = 100) and the traditional surgery group (n = 58). In the minimally invasive surgery group, patients with low inguinal cryptorchidism (n = 54) underwent transscrotal surgery, and patients with high inguinal cryptorchidism (n = 46) underwent laparoscopic surgery.

The follow-up times were one week, three months, six months and one year after the operation. Scrotal colour Doppler ultrasonography was performed six months and one year after the operation. The clinical data collected included age, weight, size of cryptorchid testis, side of cryptorchidism, operative duration, postoperative hospital stay duration, hospitalization costs and postoperative complications.

Children with inguinal cryptorchidism were included in the study. Children with recurrent inguinal cryptorchidism after surgery and/or whose parents declined participation in this study were excluded.

### Transscrotal orchiopexy

After the induction of anaesthesia, the patient was placed in the supine position with the waist slightly raised. The surgical area was routinely disinfected and draped. A transverse incision was made in the middle part of the scrotum. The skin and subcutaneous tissue were cut layer by layer. The external ring was fully exposed using a small retractor pulled upward through the incision after bluntly separating the space between the external oblique and Scarpa fascia. The aponeurosis of the external abdominal oblique muscle was cut 0.5 cm to 1.0 cm through the external ring, and the middle and lower segments of the groin were fully exposed. After pushing the testis to the area near the external ring and opening the processus vaginalis, the testicle was visible. The tissue between the spermatic cord and processus vaginalis was bluntly and sharply separated until the testis could descend to the bottom of the scrotum without tension. The processus vaginalis was transected at a high location, and the external ring orifice was reconstructed by suturing the aponeurosis of the external abdominal oblique muscle to ensure the integrity of the inguinal canal. The testis was sutured in an interrupted manner to the dartos fascia of the scrotum with no torsion. The incision was closed by 5–0 absorbable sutures.

### Laparoscopic orchiopexy

After the induction of general anaesthesia, the patient was placed in the supine position.Then pneumoperitoneum (8–10 mmHg) was established. A 5-mm trocar was placed in the umbilical area, and 3- or 5-mm trocars were placed bilaterally on the midclavicular line, slightly infraumbilical. A laparoscope was introduced into the abdominal cavity to observed the internal ring area. The peritoneum of the internal ring was incised to transect the patent processus vaginalis lateral to the spermatic vessels and medial and distal to the vas deferens, enabling the vas deferens and testicular vessels to be separated. The peritoneum was removed at the anterior surface of the spermatic vessels, and the distal triangle of the peritoneum between the vessels and the vas deferens was spared. Next, the testis was pulled into the abdominal cavity. The processus vaginalis was incised distally, and the gubernaculum was identified, grasped, and divided. A 5-mm trocar was inserted from the scrotum to under the inferior epigastric vessels through the external ring. A laparoscopic clamp was introduced through the scrotal cannula and used to grasp the gubernaculum. The testis were delivered with a gentle twisting motion. After the testis was transferred into the scrotum, the spermatic vessels and vas deferens were visualized at the inguinal ring to rule out iatrogenic torsion of the vessels. When the length of the spermatic cord was sufficient to achieve tension-free placement of the testis within the scrotum, the lateral endoabdominal bands did not need to be divided; therefore, the impact on the blood supply to the testis was reduced. The testis was sutured in an interrupted manner to the dartos fascia of the scrotum. The incision was closed by 5–0 absorbable sutures.

### Transinguinal orchiopexy

Orchiopexy via the transinguinal approach.

### Statistical analysis

Continuous data are presented as the mean ± standard deviation and range. Clinical parameters between the two groups were compared with the independent samples t-test. The χ2 or Fisher test was used to categorize variables. A *p* value of < 0.05 was defined a statistically significant.

## Results

The preoperative clinical data of all patients are shown in Table 1. There was no significant difference in age, weight, or size of cryptorchid testis or side of cryptorchidism.

**Table 1:** Comparison of preoperative, intraoperative and postoperative data between the two groups

Item	Minimally invasive surgery group	Traditional surgery group	P value
Number	100	58	
Age (year)	1.64 ± 1.37	1.87 ± 1.65	0.562
Weight (kg)	11.56 ± 2.34	13.59 ± 2.53	0.642
Size of cryptorchid testis (cm)	0.78 ± 0.22	0.76 ± 0.26	0.886
Unilateral/Bilateral	78/22	43/15	
Side of cryptorchidism			
Low inguinal testes	54	32	0.887
High inguinal testes	46	26	
Patent processus vaginalis	20	9	0.483
Operative time (min)			
Low inguinal testes	26.34 ± 6.12	38.73 ± 8.84	0.033
High inguinal testes	51.83 ± 11.65	49.69 ± 10.36	0.658
Postoperative hospital stay time (d)	1.38 ± 0.61	1.45 ± 0.63	0.921
Hospital costs (1000 RMB)	7.22 ± 1.51	6.94 ± 1.45	0.662

The hospital costs refers to all expenses during hospitalization

All patients in the minimally invasive surgery group underwent successful surgery, and there were no cases of conversion to conventional surgery. For patients with low inguinal cryptorchidism, the operative duration in the minimally invasive surgery group was significantly shorter than that in the traditional surgery group (*P* < 0.05). For patients with high inguinal cryptorchidism, there was no difference between the two groups in the operative duration (*P* > 0.05). Additionally, there was no significant difference in the postoperative hospital stay duration or hospital costs (*P* > 0.05) (Table 1).

There were no cases of testicular atrophy, testicular retraction, inguinal hernia or hydrocele between the two in either group. There was no significant difference in the incidence of poor wound healing between the two groups (*P* > 0.05). Although there was no significant difference in the incidence of scrotal hematoma between the two groups, the incidence in the minimally invasive surgery group was higher than that in the traditional surgery group (9% vs 3.4%). In the minimally invasive surgery group, scrotal hematoma occurred mostly in patients who underwent surgery with a transverse scrotal incision for orchiopexy, of which there were 8 cases in total (Table 2).

**Table 2:** Comparison of postoperative complications between the two groups

Item	Minimally invasive surgery group	Traditional surgery group	P value
Number	100	58	
Testicular atrophy	0	0	
Testicular retraction	0	0	
Inguinal hernia	0	0	
Hydrocele	0	0	
Poor wound healing	5 (5.0%)	4 (6.8%)	0.620
Scrotal haematoma	9 (9.0%)	2 (3.4%)	0.186

All patients were followed up for more than 12 months, with a median follow-up time of 14 months. One year after the operation, the size of cryptorchid testis was 1.30 ± 0.26 cm in the minimally invasive surgery group and 1.26 ± 0.31 cm in the traditional surgery group, indicating improvement compared with before the operation; however, there was no significant difference between the two groups (P = 0.921).

## Discussion

Cryptorchidism can affect reproductive function, cause testicular torsion and increase the probability of testicular tumours [15, 16]. The chance of self-descent of testis after 6 months is obviously reduced. Therefore, children with cryptorchidism require evaluation for the medical intervention after 6 months [17–19].

The currently available methods for inguinal cryptorchidism are the transinguinal incision orchiopexy, the transcrotal incision orchiopexy and the laparoscopic orchiopexy. The traditional transinguinal approach involves external oblique muscle incision, dissection of spermatic vessels and vas deferens, high transection or ligation of the processus vaginalis and orchiopexy in the scrotum. This surgical procedure provides clear exposure and is technically mature and effective; however, this surgery will leave obvious scars in the inguinal area. With the increasing demands of the aesthetics, paediatric surgeons need to consider not only the surgical effect but also the aesthetics of surgery.

The length of the inguinal canal in a 1-, 2- and 3-year-old child is 1.4 cm, 1.9 cm, and 2.7 cm, respectively, and most children with cryptorchidism have a shorter inguinal canal than other children [20]. Therefore, transcrotal approach allows transection or ligation of the patent processus vaginalis and dissection of the vas and vessels up to the level of the external inguinal ring, which is usually sufficient for low inguinal testes. Then, the testis can be placed in the scrotum without tension. This operation is simple and leads to less trauma and postoperative pain, and the incision is located in the scrotal fold with a good cosmetic effect. In this study, we performed this procedure in children with low inguinal cryptorchidism, and the operative duration of this method was significantly shorter than that of the traditional surgery (*P* < 0.05). However, the scrotal incision is low and small and provides a relatively small surgical field. The difficulty of the operation lies in fully releasing the spermatic cord vessels and achieving high ligation or transection of the processus vaginalis. In patients with a high testicular position or older age, the operation is more difficult [21]. Therefore, we chose to apply this operation in cases of low inguinal cryptorchidism. If the spermatic cord vessels could not be fully released and the processus vaginalis could not be ligated or transected, the external ring could be incised at 0.5–1.0 cm to fully release the spermatic cord and ligate or transect the processus vaginalis. In this group, the external ring was incised in 5 cases, and good results were achieved. Therefore, although orchiopexy through a scrotal incision has the advantages of a short operative duration and good cosmetic effect, its indications should be well understood prior to the operation.

For patients with high inguinal cryptorchidism, laparoscopic orchiopexy was used. This surgical procedure involves no inguinal incision, thus results in a good cosmetic effect. Additionally, the surgical field of the laparoscopic exploration is large and clear, allowing determination of the presence and location of the testis (especially in high cryptorchidism). The surgery can be carried out under direct vision, thus reducing damage to the testicular blood supply and ensuring tension-free testicular descent into the scrotum. Although laparoscopic surgery carries the risk of complications, such as intestinal injury, bladder injury and subcutaneous emphysema caused by CO_2_ pneumoperitoneum, these complications have a low incidence and can be avoided if care is taken during the operation [22, 23].

High inguinal cryptorchidism can easily occur with patent processus vaginalis. Due to the difficulty of high ligation, hydrocele or inguinal hernia as complications after surgery are common concerns. A study by Ceccanti S et al. reported that high ligation of the processus vaginalis was not performed for a high-traversing processus vaginalis and that this procedure did not increase the risk of an indirect inguinal hernia or hydrocoele [24]. Handa R et al. demonstrated that the absence of ligatures or sutures in the inner ring orifice during laparoscopic orchiopexy also did not increase in the risk of an indirect hernia or hydrocoele [25]. At our centre, we also did not perform high ligation of the processus vaginalis in cases of a high-traversing processus vaginalis, and there were no cases of hydrocele or inguinal hernia as complications in this study.

Scrotal hematoma was a common postoperative complication that occurred mostly in children who underwent transcrotal orchiopexy in this study. The transcrotal procedure causes greater damage to the scrotum, possibly due to traction, and therefore may increase the risk of postoperative scrotal hematoma. Therefore, the operation should be performed gently. Attention should be paid to haemostasis and application of pressure on the scrotum after surgery to reduce the occurrence of scrotal hematoma. During the perioperative period and 1 year follow-up time, there were no cases of testicular atrophy, testicular retraction, inguinal hernia or hydrocele in either group, and the improvement of the size of cryptorchid testis was similar, which indicated that the minimally invasive surgery and traditional surgery had good and similar clinical effects.

This single-centre retrospective study had a small sample size and short follow-up time. Multi-centre, large-sample, medium and long-term follow-up studies need to be completed to determine the clinical outcomes of these procedures more objectively.

## Conclusion

Minimally invasive surgery including surgery with transscrotal incision for low inguinal cryptorchidism and laparoscopic surgery for high inguinal cryptorchidism is as safe and effective as traditional surgery and provides good cosmetic effects.

## Data Availability

The datasets of the current study are available from the corresponding author upon reasonable request.

## References

[1] Berkowitz GS, Lapinski RH, Dolgin SE (1993). Prevalence andnatural history of cryptorchidism. Pediatrics.

[2] Murphy F, Paran TS, Puri P (2007). Orchiopexy and its impact onfertility. Pediatr Surg Int.

[3] Annandale T (1879). Case in which a testicle congenitally displaced into the perinaeum was successfully transferred to the scrotum. Br Med J.

[4] Ang CW, Forrest J (2008). Diagnostic laparoscopy and management of the impalpable testis-a review of 10 years’ practice at a non-paediatric specialist centre. J Pediatric Urol.

[5] He D, Lin T, Wei G (2008). Laparoseopic orchiopexy for treating inguinal canalicular palpable undescended testis. J Endourelogy.

[6] Riquelme M, Aranda A, Rodriguez C (2006). Laparoscopie or chiopexy for palpable undeseended testes:a five-year experience. J Laparoendoscopic Adv Surg Tech A.

[7] Denes FT, Saito FJ, Silva FA (2008). Laparoseopic diagnosis and treatment of nonpalpable testis. International Braz J Urol.

[8] Yang Z, Li S, Zeng H (2020). Laparoscopic orchiopexy versus open orchiopexy for palpable undescended testis in children: a prospective comparison study. J Laparoendosc Adv Surg Tech A.

[9] Elderwy AA, Kurkar A, Abdel-Kader MS (2014). Laparoscopic versus open orchiopexy in the management of peeping testis: a multi-institutional prospective randomized study. J Pediatr Urol.

[10] Escarcega-Fujigaki P, Rezk GH, Huerta-Murrieta E (2011). Orchiopexy-laparoscopy or traditional surgical technique in patients with an undescended palpable testicle. J Laparoendosc Adv Surg Tech A.

[11] Bianchi A, Squire B (1989). Transscrotal orchidopexy:orchidopexy revised. Pediatr Surg Int.

[12] Takahashi M, Kurokawa Y, Nakanishi R (2009). Low transscrotal orchidopexy is a safe and effective approach for undeseended testes distal to the external inguinal ring. Urol Int.

[13] Dayanc M, Kibar Y, Irkilata HC (2007). Long-term outcome of scrotal incision orchiopexy for undescended testis. Urology.

[14] Callewaert PR, Rahnama'i MS, Biallosterski BT (2010). Scrotal approach to both palpable and impalpable undescended testes:should it become our first choice?. Urology.

[15] Hutson JM, Balic A, Nation T (2010). Cryptorchidism. Semin Pediar Surg.

[16] Lip SZ, Murchison LE, Cullis PS (2013). A meta-analysis of the risk of boys with isolated cryptorchidism developing testicular cancer in later life. Arch Dis Child.

[17] Ritzén EM, Bergh A, Bjerknes R (2007). Nordic consensus on treatment of undeseended testes. Acta Paediatr.

[18] Niedzielski JK, Oszukowska E, Stowikowska-Hilczer J (2016). Undescended testis·current uends and guidelines:a review of the literature. Arch Med Sci.

[19] Gapany C, Frey P, Cachat F (2008). Management of cryptorchidism in children:guidelines. Swiss Med Wkly.

[20] Xu T, Zhu DL, Zhong J (2003). Anatomical and clinical features of indirect inguinal hernia in boys. Journal of Practical Pediatrics Clinical.

[21] Al-Mandil M, Khoury AE, El-Hout Y (2008). Potential complications with the prescrotal approach for the palpable undescended testis? A comparison of single prescrotal incision to the traditional inguinal approach. J Urol.

[22] Pelizzo G, Bernardi L, Carlini V (2017). Laparoscopy in children and its impact on brain oxygenation during routine inguinal hernia repair. J Minim Access Surg.

[23] Hsieh MH, Bayne A, Cisek LJ (2009). Bladder injuries during laparoscopie orehiopexy:incidence and lessons learned. J Urol.

[24] Ceccanti S, Zani A, Mele E (2014). Orchidopexy without ligation of the processus vaginalis is not associated with an increased risk of inguinal hernia. Hernia.

[25] Handa R, Kale R, Harjai MM (2005). Laparoscopic orchiopexy: Is closure of the internal ring necessary?. J Postgrad Med.

